# Evaluating inter- and intra-rater reliability in assessing upper limb compensatory movements post-stroke: creating a ground truth through video analysis?

**DOI:** 10.1186/s12984-024-01506-7

**Published:** 2024-12-20

**Authors:** Lena Sauerzopf, Celina G. Chavez Panduro, Andreas R. Luft, Benjamin Kühnis, Elena Gavagnin, Tim Unger, Christopher Easthope Awai, Josef G. Schönhammer, Jürgen Degenfellner, Martina R. Spiess

**Affiliations:** 1 Institute of Occupational Therapy, ZHAW School of Health Sciences, Winterthur, Switzerland; 2 Faculty of Medicine, University of Zurich, Zurich, Switzerland; 3 University for Continuing Education Krems, Krems, Austria; 4 Neuroscience of Motivation and Cognition in Rehabilitation (NeuroCoRe) Lab University of Zurich and University Hospital Zurich, Zurich, Switzerland; 5 Institute of Business Information Technology, ZHAW School of Management and Law, Winterthur, Switzerland; 6 Centre for Artificial Intelligence, ZHAW School of Engineering, Winterthur, Switzerland; 7 Data Analytics and Rehabilitation Technology (DART) Lab, Lake Lucerne Institute, Vitznau, Switzerland; 8 Rehabilitation Engineering Laboratory, ETH Zurich, Zurich, Switzerland; 9 Institute of Physiotherapy, ZHAW School of Health Sciences, Winterthur, Switzerland

**Keywords:** Stroke, Upper extremity, Compensatory movements, Machine learning, Ground truth, Inter-rater reliability, Intra-rater reliability

## Abstract

**Background:**

Compensatory movements frequently emerge in the process of motor recovery after a stroke. Given their potential for unfavorable long-term effects, it is crucial to assess and document compensatory movements throughout rehabilitation. However, clinically applicable assessment tools are currently limited. Deep learning methods have shown promising potential for assessing movement quality and addressing this gap. A crucial prerequisite for developing an accurate measurement tool is ensuring reliability in assessing compensatory movements, which is essential for establishing a valid ground truth.

**Objective:**

The study aimed to assess inter- and intra-rater reliability of occupational and physical therapists’ visual assessment of compensatory movements based on video analysis.

**Methods:**

Experienced therapists evaluated video-recorded performances of a standardized drinking task through an online labeling system. The standardized drinking task was performed by seven individuals with mild to moderate upper limb motor impairments after a stroke. The therapists rated compensatory movements in predetermined body segments and movement phases using a slider with a continuous scale ranging from 0 (no compensation) to 100 (maximum compensation). The collected data were analyzed using a generalized-linear mixed effects model with zero-inflated beta regression to estimate variance components. Intraclass correlation coefficients (ICC) were calculated to assess inter- and intra-rater reliability.

**Results:**

Twenty-two therapists participated in this study. Inter-rater reliability was good for the phases of reaching, drinking, and returning (ICC ≥ .0.75), and moderate for both phases of transporting. Intra-rater reliability was excellent for the drinking phase (ICC > 0.9) and moderate to good for the phases of reaching, transporting, and returning of our cohort. ICCs for smoothness and interjoint coordination were poor for both inter- and intra-rater reliability. The data analysis unveiled a wide range of credible intervals for the ICCs across all domains examined in this study.

**Conclusions:**

While this study shows promising inter- and intra-rater reliability for the drinking phases within our sample, the wide credible intervals raise the possibility that these results may have occurred by chance. Consequently, we cannot recommend the establishment of a ground truth for the automatic assessment of compensatory movements during a drinking task based on therapists’ ratings alone.

**Supplementary Information:**

The online version contains supplementary material available at 10.1186/s12984-024-01506-7.

## Background

The Global Burden of Disease Study 2019 shows an increase in overall stroke incidents and exposes stroke as the third-leading cause of death and disability combined. About 80% of stroke survivors are affected by motor deficits, with approximately 50% experiencing persistent upper limb impairments [17, 27, 42].

Considering the involvement of arm and hand functions in activities of daily living (ADL), facilitating upper limb motor recovery following a stroke is essential. According to the current state of scientific consensus, motor recovery is distinguished by true recovery and motor compensation [8, 24, 32]. The Stroke Recovery and Rehabilitation Roundtable (SRRR) defines true recovery as regaining the same movement patterns as available before the injury and motor compensation as the development of new motor patterns by using intact body structures to accomplish an activity goal [8]. The definitions provided by Levin et al. [32] and Kleim [24] distinguish between motor compensation and true recovery across three different levels of the international Classification of Functioning, Disability and Health (ICF [62]) domains: health condition, body function/structure and activity. According to Levin et al. [32] changes at the Health Condition level occur through processes at the neuronal level. On this level, motor compensation is characterized by structural reorganization within the brain, where other brain regions assume the functions of damaged areas. At the body function/structure level, compensation is reflected in alternative movement patterns. At the activity level, compensation is evident when different limbs or end effectors are used compared to the premorbid status. Achieving a desired goal with the impaired arm can promote its use and prevent learned non-use, potentially enhancing functional capacity and independence in activities of daily living [21, 22]. However, compensatory movements can lead to musculoskeletal changes, increasing the risk of chronic pain [12, 13, 32]. Furthermore, compensation with the unimpaired limb can negatively impact neural reorganization, potentially hindering the true recovery of the impaired limb [22, 46]. Therefore, it is important to support the true recovery on the body function/structure level during rehabilitation, rather than allowing alternative movement patterns, to minimize adverse side effects. However, to facilitate the identification and treatment of motor compensation, a comprehensive assessment is necessary [10, 26]. Compensatory movements can be assessed by measuring the quality of upper limb movement patterns through motion analysis. Possible approaches to conduct this analysis include qualitative descriptions of movements based on visual observation, observer-based scoring using standardized assessment tools or kinematic motion analysis technologies, such as marker-based motion capture systems [50].

In research settings, marker-based motion capture systems are considered the gold standard for motion analysis. Unfortunately, its implementation in clinical practice is only possible to a limited extent because of high costs, duration of the assessment as well as requirement for high-quality instruments and specialized training [60].

Core measurement sets recommended to be applied in motor rehabilitation and recovery trials post-stroke describe the Fugl-Meyer Assessment (FMA, [16]) as essential tool for evaluating upper limb body functions, and the Action Research Arm Test (ARAT, [33]) for assessing motor function at the activity level [25, 41]. However, previous research indicated that these assessments simply focus on the ability to complete a task, without capturing the movement quality. Hence they do not differentiate between the recovery of movement patterns and the use of compensatory movement strategies [26, 32, 44]. In response to the need for an assessment specifically designed to measure motor compensation, Levin et al. [30] developed the Reaching Performance Scale for Stroke (RPSS). The RPSS [30] is an ordinal-scale assessment tool that evaluates compensatory movements of the upper extremity during two specific reaching tasks, excluding drinking motions. Recent studies by Subramanian et al. [54, 55] on the RPSS [30] provide promising results, describing it as a valid, reliable, and responsive scale for visually assessing motor compensation. However, the assessment is susceptible to ceiling effects due to its ordinal scaling. To our knowledge the RPSS is not yet commonly used in clinical practice. Consequently, therapists assess compensatory movements qualitatively through visual observation relying on their clinical expertise and experience [14, 28, 44, 47].

In recent years, considerable research has focused on developing assessments for motor compensation. However, due to the complexity of developing accurate and sensitive observer-based scoring assessments and the lack of consensus regarding the use of marker-based motion capture systems, further research is required to construct reliable, feasible and affordable measurement tools [26, 29, 47, 51].

Previous research has demonstrated that kinematic motion analysis technologies, such as marker-based motion capture systems, wearable sensors, marker-free vision sensors (e.g. simple cameras, or Microsoft Kinect depth sensors) or sensors embedded in rehabilitation training systems, provide objective, consistent, and precise detection of compensatory movements through automated processes, devoid of any floor and ceiling effects respectively [23, 28, 36, 60]. Recent reviews [39, 47] have highlighted the advantages of Machine Learning (ML) algorithms, which include high agreement levels, high accuracy, and low-cost, unobtrusive home based monitoring of patients. Supervised ML algorithms depend on labeled data, which serves as ground truth, to derive an optimal model capable of accurately predicting outcomes or classifying new, unseen data by leveraging learned patterns from the training dataset [5, 40]. In stroke rehabilitation, a promising domain for applying ML algorithms lies in the automated analysis of movement patterns in impaired limbs [39].

Therefore, we are currently conducting larger project to develop a ML-supported tool to analyze compensatory movements of the upper extremities and trunk on the body function/structure level, which can be used remotely with simple methods in the homes of patients after stroke [52, 56]. In this larger study, we plan to utilize simple webcam or smartphone cameras to assess compensation during a drinking task [1]. During the development of the ML algorithm, the completion of the task is recorded with a webcam and manually labeled by experienced therapists, who assess the extent of compensatory movements. This data was intended to be used to create a ground truth for the ML algorithm. While therapists are trained to assess movement quality in person (3-dimensional), it is unclear if they can do so when rating a drinking task based on videos (2-dimensional). The findings from Martinez et al. [35], Bernhardt et al. [6] and Bernhardt et al. [7] demonstrated good reliability in assessing reaching and object-lifting tasks using video analysis. However, these studies did not specifically evaluate the drinking task.

We seek to imitate human decision-making behavior using ML-supported models. To train an ML algorithm to imitate human intelligence, i.e. to build a ground truth that reflects human knowledge, we must make implicit therapeutic decisions, such as rating compensatory movements, explicitly available. This raises a broader discussion, which we intend to explore in this article. If we want ML to learn from human intelligence, a key challenge is determining whether humans can reliably assess movements during a drinking task from 2-dimensional video recordings in the first place. Depending on the findings, it remains uncertain whether basing a ground truth for assessment on human decisions would be a viable or effective approach.

Therefore, we conducted this study to investigate the inter-rater and intra-rater reliability of experienced therapists’ assessment of compensatory movement behavior based on video recordings and hence infer if such ratings are suitable for creating a ground truth for an ML application.

## Methods

To recruit experienced occupational and physical therapists as raters, we reached out to outpatient clinics, hospitals, and rehabilitation centers via E-mail using snowball sampling. Additionally, we directly approached colleagues within our network of contacts. All raters (1) were licensed occupational or physical therapists, (2) who have at least two years of experience treating post-stroke patients and (3) were clinically active in the field of neurology at the time of study inclusion. The raters provided their informed consent electronically via REDCap (Research Electronic Data Capture) [18, 19]. We followed the data protection regulations of Switzerland and the ethical guidelines by the local Ethics Northwest and Central Switzerland (BASEC-Nr. Req-2023-01147) regarding data collection, data transmission, storage, and data analysis.

### Video recordings

In this project, we were using secondary video data from the mentioned larger research project entitled “Tele-Assessment: Leveraging Deep Learning to Assess Upper Limb Kinematics after Stroke with Off-the-shelf Webcams” [52] that some members of this project team were also involved in. Specifically, utilized video data from seven individuals who had experienced a stroke and had given informed consent for the anonymized use of their video data in research projects. To ensure anonymity the persons’ faces have been blurred (“pixelated”), and the videos have been used without sound to prevent voice recognition. In the larger research project individuals after stroke (1) were included if they were over 18 years, (2) had a verified stroke diagnosis, (3) were in the subacute or chronic phase (4) demonstrated at least a partial ability in executing a reaching and drinking motion involving a cup, (5) and have given their written consent to participate in the study. Individuals after stroke were excluded if they were diagnosed with other neurological diseases or had other upper limb impairments, such as orthopedic restrictions. These persons were recruited from the cereneo clinic and the University Hospital Zurich Stroke Registry. Table 1 provides an overview of the characteristics of the patients displayed in the seven included videos [52].

**Table 1:** Demographics of persons with stroke appearing the seven included videos

Included Recruited sample, *n*	*7*
Gender	
Female, *n* (%)	*2* (28.6)
Male, *n* (%)	*5* (71.4)
Age in years, mean (SD)	67.14 (13.90)
Time poststroke in weeks, mean (SD)	67.53 (57.04)
More affected side	
Right, *n* (%)	*3* (42.9)
Left. *n* (%)	*3* (42.9)
Both *n* (%)	*1* (14.2)
FMA-UE (0–66), mean (range)	56.57 (46–66)
BBT, mean (range)	40.86 (15–64)
MI (0–100), mean (range)	90.86 (72–100)

*SD* standard deviation, *FMA-UE* Fugl-Meyer Assessment (Upper Extremity subtest), *BBT* Box and Block Test, *MI* Motricity Index

The included individuals in the larger study project were asked to conduct a standardized drinking task according to Alt Murphy et al. [1]. For our study, we selected one video sequence from a total of 40 trials per patient. Each of the selected video sequences show a single execution of the drinking task.

### Measurement procedure and camera set-up

As mentioned above, we used the secondary data from the main project [52]. For the recordings, an optical motion capture system was employed, using technologies from Optitrack, Qualisys and Vicon (sampling frequency of 100 Hz). In the main project, we tested three camera angles (frontal, 45 degrees left, 45 degrees right) to identify the optimal angle for movement observation. The detailed setup is described in the study of Unger et al. [59].

**Fig. 1:**
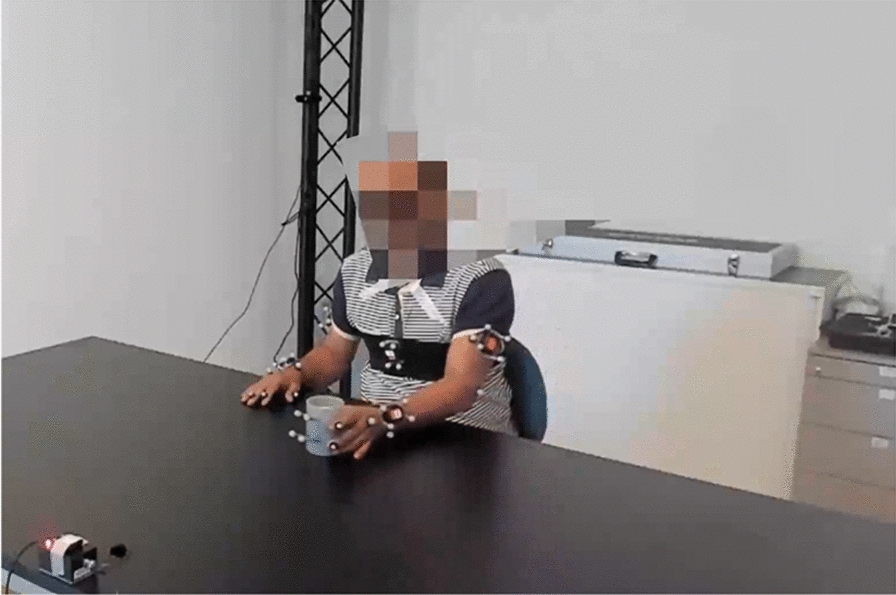
Measurement Set up

An example of the measurement setup is shown in Fig. 1.

For this reliability study, we used a single camera positioned at an ipsilateral 45-degree angle to the subject, (e.g., to observe the left arm, the camera was placed on the left side of the participant). We determined that a 45-degree angle was most effective for assessing movements in the frontal and sagittal planes. Due to the pixelation of participant’s faces in the videos for data protection reasons, the wrist and the hand are only partially visible during the drinking phase. All other joints on the observed side remain visible.

### Procedure

We stored and managed the video recordings and the labeling system (for details see Labeling System) using REDCap [18, 19] hosted at Zurich University of Applied Sciences (ZHAW), Switzerland. REDCap is a secure, web-based software platform designed to support data capture for research studies. After obtaining informed consent, each rater was granted access to the labeling system via a personalized link. The study information provided to raters during the informed consent process included a thorough description of the rating procedure. We also provided a written manual containing detailed information about the program REDCap, the labeling process, and instructions on all aspects of the labeling system’s application (e.g. how to assess compensatory movements, what “not assessable” means). The manual provides descriptions of potential compensatory movements for each body region and movement direction to be assessed within the labeling system. The manual (in German) is available upon request. Each rater observed and labeled seven videos of seven individuals post-stroke with varying levels of motor impairments in the upper extremities, at two different time points. An example video is provided in the supplementary material. The raters had the flexibility to conduct the video analyses independently of time and place, with the option to take breaks using a generated return code. We anticipated that assessing the seven videos in the first labeling round would take approximately one hour. Two researchers were available by email for any queries or difficulties. After completing the first labeling round, raters received an automatic electronic message request after two weeks to undertake the second labeling round (for intra-rater reliability). In this round, raters viewed and rated the same seven videos but in a different order. The initial version of the labeling system was tested by two trained physiotherapists from our research team and adapted with regard to comprehensibility

Our labeling system contains the activity “drinking from a glass”, as it is recommended by the SRRR for measuring recovery of the upper limb capacity [26]. We used the standardized protocol by Alt Murphy et al. [1, 2] as a guide for defining the drinking phases and corresponding movements. The so-called drinking task is described in 5 phases. The phases comprise (1) Reaching (includes grasping), (2) Forward transport (glass to mouth), (3) Drinking, (4) Back transport (glass to table, includes release of grasp), and (5) Returning (hand back to initial position) [1, 2].

Furthermore, we consulted the compensatory movement scoring checklist developed by Barth et al. [4] and the RPSS by Levin et al. [30], both designed to describe compensatory movements during upper-extremity reaching. The checklist by Barth et al. [4] is a tool that has not yet been validated for clinical purpose. It incorporates elements from existing assessments and describes compensatory behavior of the head, trunk, shoulders, elbows, forearms, wrists, and fingers, as well as describing the smoothness (fluidity) of movement. The checklist was more detailed than needed for our purpose of creating a labeling system, so we opted to adapt it instead. We selected only those items that were directly relevant to our research. Based on the checklist, the RPSS, and the five phases of the standardized drinking task, we identified relevant body segments and created our labeling system.

**Fig. 2:**
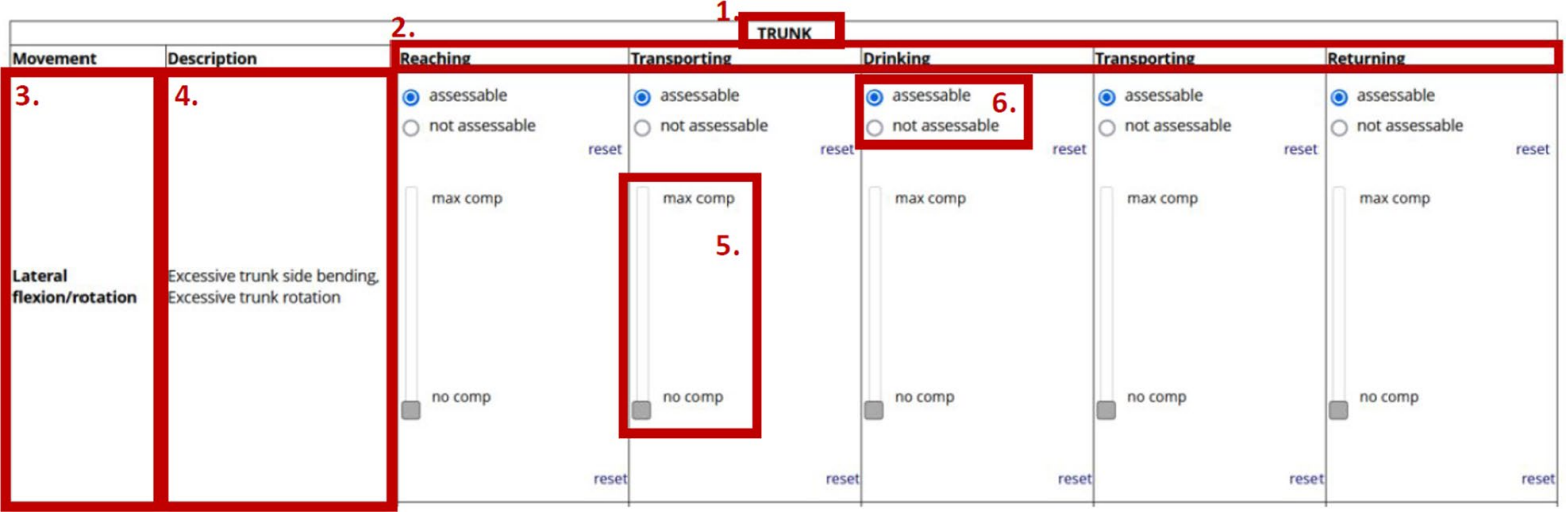
Labeling system

Figure 2 shows an excerpt of the labeling system, as well as explanations for each individual area. A detailed overview of all body regions and movement directions evaluated using the labeling system is provided in Supplemental Material 1. Additionally, a German version of this table was provided to the therapists in the written manual. In Supplemental Material 2, we propose adjustments to the description of compensatory movement patterns based on expert’s feedback received after data collection throughout the course of this project.

1. **Body region:** The top line of each section indicates the body region to be observed. The following six body regions were incorporated: trunk, shoulder, elbow, forearm, wrist, and fingers. In addition, we capture the section “global” which involves assessing the overall impression of smoothness (movement flow) and interjoint coordination (interaction between joints).
2. **Phases of movement according to **Alt Murphy et al. [1, 2]**:** Raters assessed the amount of compensatory movement for the following five phases of arm movement during the drinking task separately:

Reaching: Reach for the cup and grasp the cup

Forward Transport: Transporting the cup from the table to the mouth.

Drinking: Drinking movement.

Back Transport: Transporting the cup back to the table and releasing the cup on the table.

Returning: The hand moves back to the starting position.

3. **Movement directions:** The movements to be assessed were the following. Trunk lateral flexion/rotation, trunk flexion, shoulder flexion, horizontal shoulder adduction/scapula elevation/abduction, elbow extension, elbow flexion, forearm pronation/supination, wrist flexion, grasp patterns/hand opening, overall smoothness, and overall interjoint coordination. Each movement was clearly defined to the raters in a short text.
4. **Description:** A specification of the movements and examples of compensation movements are described in the second column. For example, the lateral flexion/rotation movement of the trunk is described as a compensatory movement if excessive trunk side bending, or excessive trunk rotation is observed by the raters during the respective movement phases.
5. **Rating of the compensation movements:** The participating therapists were asked to rate compensatory movements for each body segment, movement direction and phase of movement using a slider. The slider was associated with a continuous rating scale ranging from 0 to 100 (0 = no compensation; 100 = highest possible compensation).
6. **Assessability of the rating:** Additionally, the raters had the option to indicate if they were unable to assess the compensation of a movement (= not assessable) because the movement was not visible. This situation occurred, for example, when the movement was not perceptible due to face anonymization (pixelation).

### Reliability

Reliability refers to the accuracy and consistency of an assessment. It describes to what extent changes in measured values can be attributed to actual changes in the observed object rather than to normal fluctuations or measurement errors [53]. Reliability is distinguished between Inter-Rater Reliability and Intra-Rater Reliability. Inter-Rater Reliability assesses the degree of agreement between measurements made by different raters. Intra-Rater Reliability examines the consistency of measurements taken by the same rater at different times [37].

### Statistical analysis

For an expected reliability in ICC of 0.85 based on a two-way ICC Model [9], a confidence interval width of 0.3, a significance level alpha of 0.05 and seven video files that are assessed exactly twice by each therapist, the number of therapists must be 20, including a redundancy for drop-out of 25%, the number of participants is 25.

Due to the nature of the observed data, which were non-normal, skewed and zero-inflated, ANOVA-based ICCs which rely on normality assumptions could not be used. We estimated a generalized-linear mixed effects model with zero-inflated beta regression to estimate variance components instead (see Supplementary Material 3 for statistical details).

We calculated intraclass correlation coefficients (ICCs) for each drinking phase (averaged over all body segments and movement directions) as well as the overall score for all phases regarding smoothness and interjoint coordination to assess inter-rater reliability and intra-rater reliability. Bayesian credible intervals (CI) indicate a 95% probability that the true estimate lies within the interval [20]. We applied this approach to each phase of the drinking task as well as across all phases for smoothness and interjoint coordination.

For all statistical analysis we used R [45]. R-Codes are available on request from the corresponding author.

In categorizing the ICCs, values below 0.5 were considered indicative of poor reliability, values between 0.5 and 0.75 were considered indicative of moderate reliability, values between 0.75 and 0.90 indicated good reliability, and values above 0.90 indicated excellent reliability [43].

## Results

Twenty-two raters with a professional background in occupational therapy (n = 15) or physical therapy (n = 7), and a mean of 10.34 ± 8.07 years of professional experience in neurology participated in this study. Most of the participating therapists worked in inpatient rehabilitation (n = 9). An overview of therapists’ characteristics is provided in Table 2.

**Table 2:** Therapists’ characteristics

Included Sample, *n*	*22*
Professional Group	
OT,* n* (%)	*15* (68.2)
PT,* n* (%)	*7* (31.8)
Gender	
Female, *n* (%)	*18* (81.8)
Male, *n* (%)	*4* (18.2)
Age in years, mean (SD)	35.32 (9.09)
Work Setting^a^	
Inpatient acute care g *n* (%)	*5* (22.7)
Inpatient Rehabilitation *n* (%)	*9* (40.9)
Outpatient treatment *n* (%)	*8* (36.4)
Home-based therapy *n* (%)	*5* (22.7)
Tele-Therapy *n* (%)	*1* (4.5)
Professional Experience	
Years since basic clinical training was completed, mean (SD)	11.77 (8.13)
Years of experience in the field of neurology, mean (SD)	10.34 (8.07)

*PT* physiotherapy, *OT* occupational therapy, *SD* standard deviation

^a^Multiple selections were possible

Initially, a total of 27 therapists agreed to participate in our study. Participant dropout (n = 5) occurred due to two reasons: two persons initiated the first labeling round but did not complete it, and three persons completed the first labeling round but abstained from the second. All therapists who dropped out of the study received at least one email reminder.

### Descriptive statistics

Per rater and video, we collected 55 scores, resulting in a total of 16,940 data points. In the first round, 155 movements were labeled as “not assessable”, with 177 movements receiving the same label in the second round. We excluded all movements labeled as “not assessable” in either the first, second, or both labeling rounds. Ultimately, we analyzed a dataset of 8,245 measurement scores for each labeling round. Due to the large volume of data, it is not feasible to present all our findings in detail. Therefore, we focus on presenting specific results that we believe are crucial for understanding the conclusions.

**Fig. 3:**
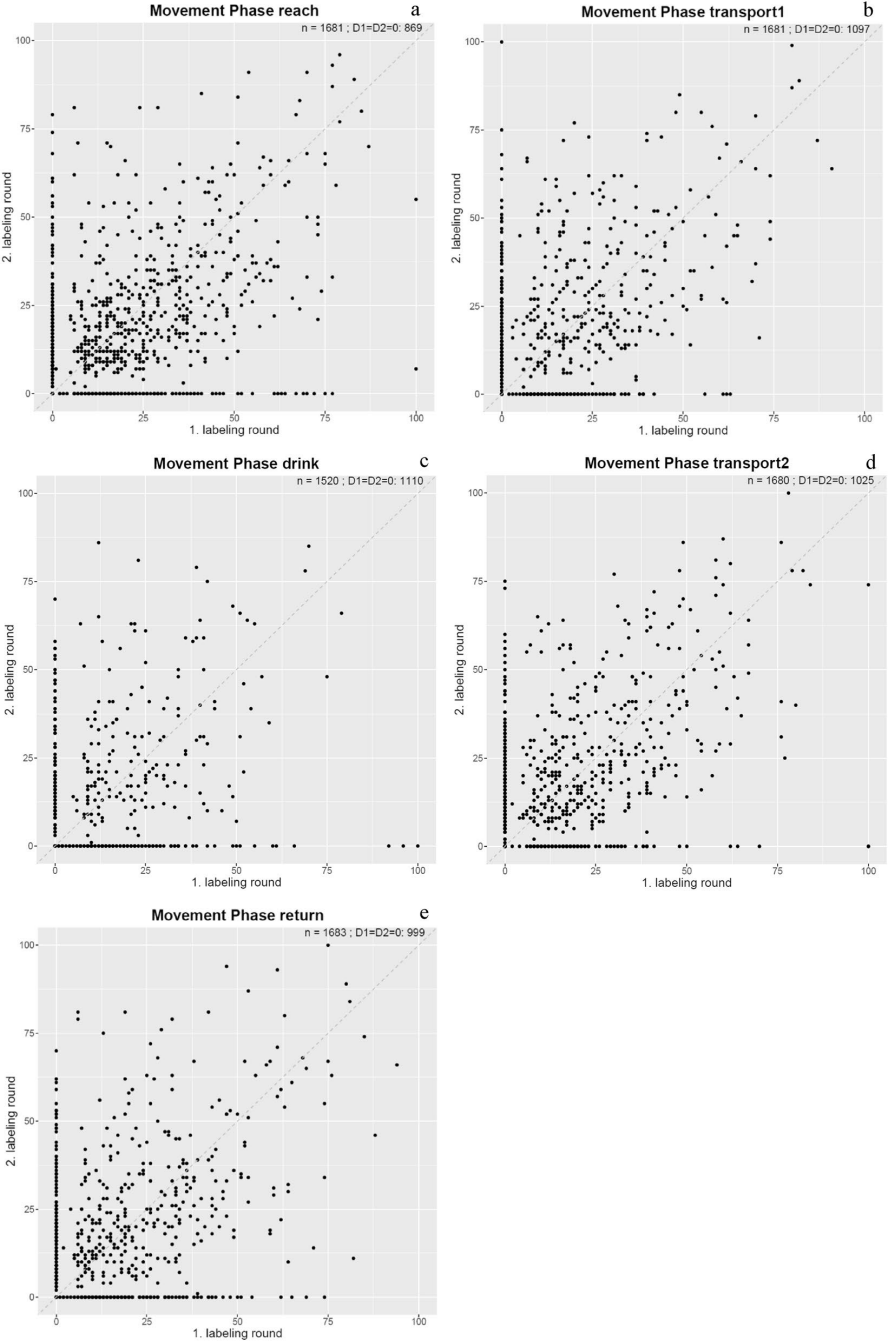
Distribution of the Raters Labeling of Compensatory Movements across each Movement Phase. n = the total number of data points presented in the scatterplot; D1 = D2 = 0: = The count of matching 0 values between the first and second labeling round

Figure 3 presents a series of scatterplots presenting all values assigned per movement phase during the first and second labeling rounds. The values represent all assessments made by the therapists when rating the movements observed in the videos. A total of 1,520 values were recorded for the drinking phase, while the remaining four phases each counted between 1,680 and 1,683 values. Among the phases, the drinking phase exhibited the highest frequency of 0 values (n = 1110) in both the first and the second labeling rounds, whereas the reaching phase showed the lowest frequency of 0 values (n = 869) in both labeling rounds. In general, the scatterplots reveal substantial variance in the rating between the first and second labeling rounds. Notable instances are observed where raters assigned a score of 0 during one labeling round but a compensation value greater than 25 during the other round. This phenomenon is illustrated by the distribution of data points along the x- and y-axes.

The table in Supplementary Material 4 presents a comprehensive summary of the characteristic values for deviations, calculated as the difference from the first to the second labeling rounds.

### Inter-rater reliability

The inter-rater reliability results for the five phases of movement during the drinking task, along with the overall ICCs for smoothness and interjoint coordination, are presented in Table 3. Inter-rater reliability was good (ICC ≥ 0.75) for the reaching (ICC of 0.76, 95% CI 0.44–1), drinking (ICC of 0.88, 95% CI 0.60–1), and returning phases (ICC of 0.75, 95% CI 0.43–1). During the forward transporting phase (ICC of 0.68, 95% CI 0.38–1) and back transporting phase (ICC of 0.65, 95% CI 0.31–1) inter-rater reliability was moderate. The CIs varied widely across the five phases of the drinking task, ranging from 0.31 to 1.

**Table 3:** Overview of inter-rater reliability results

	Reaching ICC (95% CI)	Forward Transport ICC (95% CI)	Drinking ICC (95% CI)	Back Transport ICC (95% CI)	Returning ICC (95% CI)
Average data across all movement directions and body regions	0.76 (0.44–1)	0.68 (0.38–1)	0.88 (0.60–1)	0.65 (0.31–1)	0.75 (0.43–1)
Overall ICC (95% CI) Smoothness	0.49 (0.18–1)				
Overall ICC (95% CI) Interjoint coordination	0.40 (0.13–1)				

*ICC* intraclass correlation, *CI* credible interval

However, the ICCs for the domains of smoothness (ICC of 0.49, 95% CI 0.18–1) and interjoint coordination (ICC of 0.40, 95% CI 0.13–1) across all five phases of movement were poor. Again, the CIs showed considerable variation in the domain of smoothness and interjoint coordination, ranging from 0.13 to 1.

### Intra-rater reliability

Intra-rater reliability results for the five phases of movement during the drinking task and overall ICCs for smoothness and interjoint coordination are presented in Table 4. Intra-rater reliability was excellent (ICC > 0.9) for the drinking phase (ICC of 1, 95% CI 0.24–1) and moderate to good for the reaching (ICC of 0.73, 95% CI 0.22–1), forward transporting (ICC of 0.84, 95% CI 0.20–1), back transporting (ICC of 0.67, 95% CI 0.18–1) and returning phase (ICC of 0.76, 95% CI 0.21–1). The CIs varied widely across the five phases of the drinking task, ranging from 0.18 to 1.

**Table 4:** Overview of intra-rater reliability results

	Reaching ICC (95% CI)	Forward Transport ICC (95% 8CI)	Drinking ICC (95% CI)	Back Transport ICC (95% CI)	Returning ICC (95% CI)
Average data across all movement directions and body regions	0.73 (0.22–1)	0.84 (0.20–1)	1 (0.24–1)	0.67 (0.18–1)	0.76 (0.21–1)
Overall ICC (95% CI) Smoothness	0.13 (0.02–0.86)				
Overall ICC (95% CI) Interjoint coordination	0.19 (0.03–1)				

*ICC* intraclass correlation, *CI* credible interval

The smoothness (ICC of 0.13, 95% CI 0.02–0.86) and interjoint coordination domains (ICC of 0.19, 95% CI 0.03–1) over all 5 phases of movement resulted in poor ICCs. Again, the CIs showed considerable variation in the domain of smoothness and interjoint coordination, ranging from 0.02 to 1.

## Discussion

In this study we examined the inter-rater and intra-rater reliability of experienced therapists visually assessing compensatory movements in individuals post-stroke through video analysis. Our cohort of experienced therapists demonstrated moderate to excellent reliability in evaluating specific movement directions of certain body parts during particular phases of a standardized drinking task. However, as evidenced by the results of smoothness and interjoint coordination measurements, the human judgement of more global movement characteristics demonstrated poor reliability. Furthermore, the analysis revealed wide credible intervals (CIs) for the ICCs across all domains measured in this study. This wide range of CIs indicates a degree of uncertainty in our results, which warrants careful consideration and will be discussed in more detail. Nevertheless, a significant strength of our research design is its interdisciplinary approach. This project stands out due to the collaboration between researchers in engineering and clinical fields, who integrate therapists into the development process of AI-based assessments. Therapists contribute their clinical expertise, ensuring a comprehensive and practical perspective on stroke rehabilitation development within this research project.

### Statistical analysis

Within this study, we achieved moderate to good reliability for the five phases of the drinking task in our sample. However, statistical analysis revealed a wide range of CIs (ranging 0.18–1) for all these phases. According to Bayesian statistics, our CIs indicate that our effect has 95% probability of falling within the range of 0.18–1 for intra-rater reliability and 0.31–1 for inter-rater reliability [34]. Consequently, we must consider that the moderate to good reliability results within the drinking phases may not be replicable in other cohorts of experienced therapists. Therefore, we cannot generalize our results to other study populations at this time. The sample size of this study is one factor that could explain the wide ranges of CIs. Power calculation turned out not to be straight forward for this study. According to our calculation based on Bonett [9] we determined that a sample size of 22 therapists is sufficient for conducting statistical analysis with a two-way mixed effects ANOVA Model. During data analysis, we were facing non-normal, skewed and zero-inflated data. The unpredictable characteristics of our data prevented straightforward ICC calculations with an ANOVA model, requiring us to rely on alternative models, in this case, Bayesian methods [20]. Therefore, the initial power calculation may not align with the model ultimately used for the statistical analysis. This may have resulted in the study being underpowered with respect to sample size, possibly explaining the observed wide credible intervals.

As described above, our data turned out to be non-normal, skewed, and zero-inflated. Zero-inflated data in our study means that a significant number of ratings took the value 0 (= no compensation). We assume that the zero-inflated and skewed data situation occurred through the fact that the individuals performing the drinking task have been affected by mild to moderate impairments. Therefore, a considerable amount of the ratings fell in the lower third of the scale. While we are aware of this limitation, the nature of the drinking task precluded the inclusion of individuals with severe upper limb motor impairments, as they would be unable to perform the drinking task. This underscores the challenge of obtaining a dataset of videos that encompasses sufficient scope and variability in the motor impairments of the depicted individuals, making it suitable for use as a training dataset. Assuming that the rating scale can only be trained with data from individuals post-stroke with mild to moderate impairment, the scale can only be used for this range of limitations. To enhance the applicability of the assessment, we recommend incorporating additional activity tasks suitable for individuals with moderate to severe impairments. Possible adjustments are either to include the “Can” and the “Box” items from the Wolf motor Function Test (WMFT, Wolf et al. [61]), as implemented by Martinez et al. [35], or to integrate reaching for a close and a far target, as applied in the RPSS [30].

### Establishing ground truth reliability

The main aim of our study was to investigate whether observer-based scoring could be used to build a ground truth for a deep learning model assessing compensatory movements. As described above, despite achieving moderate to good reliability for the respective drinking phases, our results have limited generalizability due to the wide range of CIs in our data. Therefore, the data collected in this project is not viable for establishing a ground truth.

We emphasize two potential approaches. The first approach addresses the training and calibration of the therapists. In this study, participating therapists were provided with a manual explaining the labeling system and guiding them through the labeling process. However, the explanations may not have been sufficiently detailed. Therefore, we provide suggestions for adapting the descriptions of compensatory movement characteristics in Supplemental Material 2. Additionally, instructions on the standardized drinking task and calibration sessions for the therapists would have promoted a consistent understanding of movement compensation. Since therapists are not accustomed to labeling movements in a two-dimensional space (videos) using the computer-based rating system, we anticipate that targeted training on technical aspects would have facilitated the labeling process. Therefore, we propose that specialized training to the therapists would possibly increase the reliability of their evaluations.

Additionally, our findings have shown poor reliability in 2-dimensional visual assessment of global movement characteristics. One possible explanation of the poor reliability of these characteristics relies to the work of Levin et al. [31] and Mennella et al. [38]. They describe the drinking task as a complex movement with great individual and context dependent variability in movement performance. Thus, achieving consistent evaluations of perceived smoothness and interjoint coordination across all drinking phases based on two-dimensional (video) information may prove too challenging for humans. The low agreement observed in the assessment of movement smoothness by humans has also been acknowledged by Levin et al. [30]. However, previous clinical studies investigating motor performance in upper limb movements using marker-based motion capture systems have identified smoothness and interjoint coordination as valid and reliable key metrics for measuring movement quality and motor recovery [11, 15, 48, 49, 57]. For this reason, these parameters should be incorporated into the assessment of stroke rehabilitation recovery. As suggested by Tomita et al. [58] and Alt Murphy et al. [3] endpoint performance or number of movement units could serve as valuable metrics for assessing inter-joint coordination and smoothness. Tomita et al. [58] recommend assessing endpoint performance metrics using low-cost motion tracking systems. Additionally, they advise validating the final version of the assessments tool with a high-quality Motion Capture System to ensure precise measurement of inter-joint coordination. Therefore incorporating kinematic motion analysis technologies into the ground truth-building process offers another possible approach for assessing global movement characteristics.

### Clinical implications

Our research project serves as an initial step in the creation of an automated movement analysis system designed to classify compensatory movements during a standardized drinking task. By involving experienced therapists from clinical settings in the user-centered development process, our aim was to incorporate therapeutic expertise and bridge the gap between scientific research and clinical practice.

Further research using the data gathered in our study aims to produce a reliable and clinically applicable assessment tool for classifying compensatory movements, thereby addressing the research gap of the main study.

Therapists in clinical practice are used to classify compensatory movement in the three-dimensional room (e.g., going around the patient, having the possibility to work hands-on). In this study we could not prove that an immediate transfer of this knowledge and clinical reasoning is possible in the two-dimensional room (visual movement analysis through 2D videos). Despite this, our effort aimed to initiate the process of making the knowledge that therapists utilize in their daily clinical practice accessible for training an ML algorithm to detect compensatory movements. We attempted to achieve this by examining each body part and its movements separately, which facilitated the visualization and understanding of the therapist's expertise.

### Limitations

In our labeling system, we see both strengths and weaknesses. We created our labeling system with a considerable number (n = 55) of labeling fields, enabling a detailed classification of compensatory movements. We perceive this comprehensive approach as a notable strength of our system. Regarding the scale of the labeling system, we observe certain limitations that may have introduced biases. We implemented a scale ranging from 0 to 100, where we defined 0 as representing no compensation and 100 as indicating the highest possible level of compensation. Given the absence of defined criteria or thresholds in the literature for what constitutes the "highest possible" compensation, we propose that participating therapists likely hold varying interpretations of this concept.

Furthermore, we asked therapists to directly rate compensation on a scale from 0 to 100. In hindsight, we propose to introduce a two-step rating process, with the initial question being dichotomized into “compensation present” or “compensation not present”. In case of “compensation present”, raters would then be asked about the severity on a scale from 1 to 100. This will allow to also analyze if individual raters agree on each question individually. In our dataset, we had a large zero-inflation, which could be hinting to the fact that raters did agree on the first question (compensation present or not). However, as we did not ask in a two-step manner, we refrained from analyzing the data in this way. We felt this would introduce a bias, as we would be treating the difference between a 0 and 1 on the rating scale vastly different than any other difference, e.g., between 23 and 24.

Further limitations emerge from technical constrains affecting the rating process. The extensive size of the labeling system hindered simultaneous display of both the video and the entire labeling system on one screen. As a result, raters had to scroll down for data entry and frequently switch between viewing the videos and recording observations. Additionally, during the drinking phase, pixelation used for anonymizing patients faces inadvertently obscures parts of the fingers and wrist. These limitations were reflected by the increased frequency of raters marking “not assessable” for finger and wrist movements within the drinking phase.

To meet the aims of the overarching project, markers were applied to the patients’ extremities and trunk, as shown in Fig. 1. With respect to the marker placement, the wrist marker could have potentially limited the accuracy of the movement assessment. Moreover, the presence of markers might have influenced the individual’s motor performance, a recognized challenge in marker-based motion analysis. For future studies, we recommend conducting the drinking task without body markers to minimize these potential confounding effects. A potential source of bias in intra-rater reliability arises from characteristics of the videos. They have been filmed in different rooms, with varying backgrounds and patients wearing their personal clothing. These factors may have benefited the raters recall of initial scores during the second labeling round. We addressed this concern by implementing a protocol involving a mandatory waiting period of at least fourteen days between the two observation dates. Additionally, we altered the sequence of video presentation from the initial to the subsequent observation date.

We cannot determine the extent to which the camera set-up influenced the ability to capture compensatory movements and their magnitude, as our resources only permitted the reliability testing of one camera setup. Ideally, future research should assess the reliability of different camera setups and compare the reliability.

## Conclusion

The observer-based visual assessment of compensatory movements via two-dimensional video analysis presented challenges. While the evaluation of specific movement phases during the drinking task indicated moderate to excellent agreement among our sample of raters, we encountered wide CIs. Based on our data, we cannot recommend that algorithms for ML-supported applications are based on therapists’ judgements without specific training and calibration activities. These findings underscore the need for further research to determine how visual assessments can be effectively integrated in the process of building a ground truth for developing automated assessments of upper limb movement quality. In our larger, original research initiative we will rely on a combination of 3D movement analysis and ratings of trained and calibrated therapists.

## Supplementary Information

Additional file1 (DOCX 265 KB)

Additional file2 (MP4 679 KB)

## Data Availability

The datasets used and/or analysed during the current study are available from the corresponding author on reasonable request.

## References

[1] Alt Murphy M, Murphy S, Persson HC, Bergström U-B, Sunnerhagen KS. kinematic analysis using 3D motion capture of drinking task in people with and without upper-extremity impairments. J Vis Exp JoVE. 2018;133: e57228. 10.3791/57228. 10.3791/57228 PMC5933268 29658937

[2] Alt Murphy M, Sunnerhagen KS, Johnels B, Willén C. Three-dimensional kinematic motion analysis of a daily activity drinking from a glass: a pilot study. J Neuroeng Rehabil. 2006;3(1):18. 10.1186/1743-0003-3-18. 16914057 10.1186/1743-0003-3-18 PMC1562432

[3] Alt Murphy M, Willén C, Sunnerhagen KS. Movement kinematics during a drinking task are associated with the activity capacity level after stroke. Neurorehabil Neural Repair. 2012;26(9):1106–15. 10.1177/1545968312448234. 22647879 10.1177/1545968312448234

[4] Barth J, Klaesner JW, Lang CE. Relationships between accelerometry and general compensatory movements of the upper limb after stroke. J Neuroeng Rehabil. 2020;17(1):138. 10.1186/s12984-020-00773-4. 33081783 10.1186/s12984-020-00773-4 PMC7576735

[5] Baştanlar Y, Özuysal M. Introduction to machine learning. In: Yousef M, Allmer J, editors. miRNomics: MicroRNA biology and computational analysis, vol. 1107. Humana Press; 2014. p. 105–28. 10.1007/978-1-62703-748-8_7. 10.1007/978-1-62703-748-8_7 24272434

[6] Bernhardt J, Bate PJ, Matyas TA. Accuracy of observational kinematic assessment of upper-limb movements. Phys Ther. 1998;78(3):259–70. 10.1093/ptj/78.3.259. 9520971 10.1093/ptj/78.3.259

[7] Bernhardt J, Bate PJ, Matyas TA. Training Novice clinicians improves observation accuracy of the upper extremity after stroke. Arch Phys Med Rehabil. 2001;82:1611–8. 11689983 10.1053/apmr.2001.25143

[8] Bernhardt J, Hayward KS, Kwakkel G, Ward NS, Wolf SL, Borschmann K, Krakauer JW, Boyd LA, Carmichael ST, Corbett D, Cramer SC. Agreed definitions and a shared vision for new standards in stroke recovery research: the Stroke Recovery and Rehabilitation Roundtable taskforce. Int J Stroke. 2017;12(5):444–50. 10.1177/1747493017711816. 28697708 10.1177/1747493017711816

[9] Bonett DG. Sample size requirements for estimating intraclass correlations with desired precision. Stat Med. 2002;21(9):1331–5. 10.1002/sim.1108. 12111881 10.1002/sim.1108

[10] Buma FE, Kwakkel G, Ramsey N. Understanding upper limb recovery after stroke. Restor Neurol Neurosci. 2013;31(6):707–22. 10.3233/RNN-130332. 23963341 10.3233/RNN-130332

[11] Buma FE, Van Kordelaar J, Raemaekers M, Van Wegen EEH, Ramsey NF, Kwakkel G. Brain activation is related to smoothness of upper limb movements after stroke. Exp Brain Res. 2016;234(7):2077–89. 10.1007/s00221-015-4538-8. 26979435 10.1007/s00221-015-4538-8 PMC4893073

[12] Carr J, Shepherd R. Neurological rehabilitation: optimizing motor performance (2nd Edition). Churchill Livingstone; 2010.

[13] Cirstea MC, Levin MF. Compensatory strategies for reaching in stroke. Brain. 2000;123:940–53. 10.1093/brain/123.5.940. 10775539 10.1093/brain/123.5.940

[14] Demers M, Levin MF. Do activity level outcome measures commonly used in neurological practice assess upper-limb movement quality? Neurorehabil Neural Repair. 2017;31(7):623–37. 10.1177/1545968317714576. 28675943 10.1177/1545968317714576

[15] Dounskaia N, Shimansky Y, Ganter BK, Vidt ME. A simple joint control pattern dominates performance of unconstrained arm movements of daily living tasks. PLoS ONE. 2020;15(7): e0235813. 10.1371/journal.pone.0235813. 32658898 10.1371/journal.pone.0235813 PMC7357763

[16] Fugl-Meyer AR, Jääskö L, Leyman I, Olsson S, Steglind S. The post-stroke hemiplegic patient: I. A method for evaluation of physical performance. Scand J Rehab Med. 1975;7:13–31. 1135616

[17] GBD 2019 Stroke Collaborators. Global, regional, and national burden of stroke and its risk factors, 1990–2019: A systematic analysis for the Global Burden of Disease Study 2019. Lancet Neurol. 2021;20:795–820. 10.1016/S1474-4422(21)00252-0. 34487721 10.1016/S1474-4422(21)00252-0 PMC8443449

[18] Harris PA, Taylor R, Minor BL, Elliott V, Fernandez M, O’Neal L, McLeod L, Delacqua G, Delacqua F, Kirby J, Duda SN. The REDCap consortium: Building an international community of software platform partners. J Biomed Inform. 2019;95: 103208. 10.1016/j.jbi.2019.103208. 31078660 10.1016/j.jbi.2019.103208 PMC7254481

[19] Harris PA, Taylor R, Thielke R, Payne J, Gonzalez N, Conde JG. Research electronic data capture (REDCap)—a metadata-driven methodology and workflow process for providing translational research informatics support. J Biomed Inform. 2009;42(2):377–81. 10.1016/j.jbi.2008.08.010. 18929686 10.1016/j.jbi.2008.08.010 PMC2700030

[20] Hespanhol L, Vallio CS, Costa LM, Saragiotto BT. Understanding and interpreting confidence and credible intervals around effect estimates. Braz J Phys Ther. 2019;23(4):290–301. 10.1016/j.bjpt.2018.12.006. 30638956 10.1016/j.bjpt.2018.12.006 PMC6630113

[21] Hylin MJ, Kerr AL, Holden R. Understanding the mechanisms of recovery and/or compensation following injury. Neural Plast. 2017;2017(7125057):12. 10.1155/2017/7125057. 10.1155/2017/7125057 PMC5415868 28512585

[22] Jones TA. Motor compensation and its effects on neural reorganization after stroke. Nat Rev Neurosci. 2017;18(5):267–80. 10.1038/nrn.2017.26. 28331232 10.1038/nrn.2017.26 PMC6289262

[23] Kanzler CM, Schwarz A, Held JPO, Luft AR, Gassert R, Lambercy O. Technology-aided assessment of functionally relevant sensorimotor impairments in arm and hand of post-stroke individuals. J Neuroeng Rehabil. 2020;17(1):128. 10.1186/s12984-020-00748-5. 32977810 10.1186/s12984-020-00748-5 PMC7517659

[24] Kleim JA. Neural plasticity and neurorehabilitation: teaching the new brain old tricks. J Commun Disord. 2011;44:521–8. 10.1016/j.jcomdis.2011.04.006. 21600589 10.1016/j.jcomdis.2011.04.006

[25] Kwakkel G, Lannin NA, Borschmann K, English C, Ali M, Churilov L, Saposnik G, Winstein C, Van Wegen EE, Wolf SL, Krakauer JW, Bernhardt J. Standardized measurement of sensorimotor recovery in stroke trials: Consensus-based core recommendations from the Stroke Recovery and Rehabilitation Roundtable. Int J Stroke. 2017;12(5):451–61. 10.1177/1747493017711813. 28697709 10.1177/1747493017711813

[26] Kwakkel G, van Wegen EEH, Burridge JH, Winstein CJ, van Dokkum LEH, Alt Murphy M, Levin MF, Krakauer JW. Standardized measurement of quality of upper limb movement after stroke: consensus-based core recommendations from the second stroke recovery and rehabilitation roundtable. Neurorehabil Neural Repair. 2019;33(11):951–8. 10.1177/1545968319886477. 31660781 10.1177/1545968319886477

[27] Langhorne P, Coupar F, Pollock A. Motor recovery after stroke: a systematic review. Lancet Neurol. 2009;8:741–54. 10.1016/S1474-4422(09)70150-4. 19608100 10.1016/S1474-4422(09)70150-4

[28] Lee MH, Siewiorek DP, Smailagic A, Bernardino A, Badia SBI. Learning to assess the quality of stroke rehabilitation exercises. In: Proceedings of the 24th International Conference on Intelligent User Interfaces, 2019; 218–228. 10.1145/3301275.3302273.

[29] Lee MH, Siewiorek DP, Smailagic A, Bernardino A, Bermúdez I Badia SB. A human-AI collaborative approach for clinical decision making on rehabilitation assessment. In: Proceedings of the 2021 CHI Conference on human factors in computing systems, 2021; 1–14. 10.1145/3411764.3445472.

[30] Levin MF, Desrosiers J, Beauchemin D, Bergeron N, Rochette A. Development and validation of a scale for rating motor compensations used for reaching in patients with hemiparesis: the reaching performance scale. Phys Ther. 2004;84(1):8–22. 10.1093/ptj/84.1.8. 14992673

[31] Levin MF, Hiengkaew V, Nilanont Y, Cheung D, Dai D, Shaw J, Bayley M, Saposnik G. Relationship between clinical measures of upper limb movement quality and activity poststroke. Neurorehabil Neural Repair. 2019;33(6):432–41. 10.1177/1545968319847969. 31072222 10.1177/1545968319847969

[32] Levin MF, Kleim JA, Wolf SL. What do motor “Recovery” and “Compensation” mean in patients following stroke? Neurorehabil Neural Repair. 2009;23(4):313–9. 10.1177/1545968308328727. 19118128 10.1177/1545968308328727

[33] Lyle RC. A performance test for assessment of upper limb function in physical rehabilitation treatment and research. Int J Rehabil Res. 1981;4(4):483. 7333761 10.1097/00004356-198112000-00001

[34] Makowski D, Ben-Shachar M, Lüdecke D. bayestestR: describing effects and their uncertainty, existence and significance within the Bayesian framework. J Open Source Softw. 2019;4(40):1541. 10.21105/joss.01541.

[35] Martinez C, Bacon H, Rowe V, Russak D, Fitzgerald E, Woodbury M, Wolf SL, Winstein C. A reaching performance scale for 2 wolf motor function test items. Arch Phys Med Rehabil. 2020;101:2015–26. 10.1016/j.apmr.2020.05.003. 32433993 10.1016/j.apmr.2020.05.003 PMC7669605

[36] Maura RM, Rueda Parra S, Stevens RE, Weeks DL, Wolbrecht ET, Perry JC. Literature review of stroke assessment for upper-extremity physical function via EEG, EMG, kinematic, and kinetic measurements and their reliability. J Neuroeng Rehabil. 2023;20:21. 10.1186/s12984-023-01142-7. 36793077 10.1186/s12984-023-01142-7 PMC9930366

[37] Mehrholz J, Thomas S, Elsner B. Beurteilung von Assessments oder Testgütekriterien. Neuroreha. 2016;08:62–7. 10.1055/s-0042-106150.

[38] Mennella C, Maniscalco U, De Pietro G, Esposito M. A deep learning system to monitor and assess rehabilitation exercises in home-based remote and unsupervised conditions. Comput Biol Med. 2023;166: 107485. 10.1016/j.compbiomed.2023.107485. 37742419 10.1016/j.compbiomed.2023.107485

[39] Mennella C, Maniscalco U, De Pietro G, Esposito M. The role of artificial intelligence in future rehabilitation services: a systematic literature review. IEEE Access. 2023;11:11024–43. 10.1109/ACCESS.2023.3236084.

[40] Menshawy A. Deep learning by example: A hands-on guide to implementing advanced machine learning algorithms and neural networks. Packt Publishing; 2018.

[41] Pohl J, Held JPO, Verheyden G, Alt Murphy M, Engelter S, Flöel A, Keller T, Kwakkel G, Nef T, Ward N, Luft AR, Veerbeek JM. Consensus-based core set of outcome measures for clinical motor rehabilitation after stroke—a Delphi study. Front Neurol. 2020;11:875. 10.3389/fneur.2020.00875. 33013624 10.3389/fneur.2020.00875 PMC7496361

[42] Pollock A, Farmer SE, Brady MC, Langhorne P, Mead GE, Mehrholz J, Van Wijck F. Interventions for improving upper limb function after stroke. Cochrane Database Syst Rev. 2014. 10.1002/14651858.CD010820.pub2. 25387001 10.1002/14651858.CD010820.pub2 PMC6469541

[43] Portney LG, Watkins MP. Foundations of clinical research: applications to practice. 3rd ed. Pearson/Prentice Hall; 2009.

[44] Prange-Lasonder GB, Alt Murphy M, Lamers I, Hughes A-M, Buurke JH, Feys P, Keller T, Klamroth-Marganska V, Tarkka IM, Timmermans A, Burridge JH. European evidence-based recommendations for clinical assessment of upper limb in neurorehabilitation (CAULIN): data synthesis from systematic reviews, clinical practice guidelines and expert consensus. J Neuroeng Rehabil. 2021;18:162. 10.1186/s12984-021-00951-y. 34749752 10.1186/s12984-021-00951-y PMC8573909

[45] R Core Team. R: a language and environment for statistical computing. R Foundation for Statistical Computing [Computer software]. 2024 [cited 2024 Jun 17]. http://www.R-project.org/.

[46] Raghavan P. Upper limb motor impairment after stroke. Phys Med Rehabil Clin N Am. 2015;26(4):599–610. 10.1016/j.pmr.2015.06.008. 26522900 10.1016/j.pmr.2015.06.008 PMC4844548

[47] Rahman S, Sarker S, Haque AKMN, Uttsha MM, Islam MF, Deb S. AI-driven stroke rehabilitation systems and assessment: a systematic review. IEEE Trans Neural Syst Rehabil Eng. 2023;31:192–207. 10.1109/TNSRE.2022.3219085. 36327176 10.1109/TNSRE.2022.3219085

[48] Raj S, Dounskaia N, Clark WW, Sethi A. Effect of stroke on joint control during reach-to-grasp: a preliminary study. J Mot Behav. 2020;52(3):294–310. 10.1080/00222895.2019.1615861. 31107178 10.1080/00222895.2019.1615861

[49] Saes M, Mohamed Refai MI, van Kordelaar J, Scheltinga BL, van Beijnum B-JF, Bussmann JBJ, Buurke JH, Veltink PH, Meskers CGM, van Wegen EEH, Kwakkel G. Smoothness metric during reach-to-grasp after stroke: part 2. Longitudinal association with motor impairment. J NeuroEng Rehabil. 2021;18(1):144. 10.1186/s12984-021-00937-w. 34560898 10.1186/s12984-021-00937-w PMC8461930

[50] Schwarz A, Bhagubai MMC, Wolterink G, Held JPO, Luft AR, Veltink PH. Assessment of upper limb movement impairments after stroke using wearable inertial sensing. Sensors. 2020;20(17):4770. 10.3390/s20174770. 32846958 10.3390/s20174770 PMC7506737

[51] Schwarz A, Kanzler CM, Lambercy O, Luft AR, Veerbeek JM. systematic review on kinematic assessments of upper limb movements after stroke. Stroke. 2019;50:718–27. 10.1161/STROKEAHA.118.023531. 30776997 10.1161/STROKEAHA.118.023531

[52] Spiess M, Luft AR, Gavagnin E, Kühnis B, Sauerzopf L, Schönhammer J, Unger T, de Spindler A, Awai Easthope C. Tele-Assessment: Leveraging Deep Learning to Assess Upper Limb Kinematics after Stroke with Off-the-shelf Webcams [Internet]. 2024 [cited 2024 May 29]. https://www.zhaw.ch/en/research/research-database/project-detailview/projektid/5110/.

[53] Streiner DL, Norman GR, Cairney J. Health measurement scales: a practical guide to their development and use. 5th ed. Oxford University Press; 2015.

[54] Subramanian SK, Baniña MC, Turolla A, Levin MF. Reaching performance scale for stroke—test-retest reliability, measurement error, concurrent and discriminant validity. PM&R. 2022;14:337–47. 10.1002/pmrj.12584. 33675151 10.1002/pmrj.12584

[55] Subramanian SK, Margolese G, Turolla A, Saposnik G, Levin MF. Responsiveness of the reaching performance scale for stroke. Arch Phys Med Rehabil. 2023;104:1588–95. 10.1016/j.apmr.2023.04.020. 37178950 10.1016/j.apmr.2023.04.020

[56] Tele-Assessment. AI Supported Analysis of Post-Stroke Arm Movements with Webcams [Video recording]. Ansich. 2024 [cited 2024 Jul 11]. https://www.youtube.com/watch?v=q14Ux_F_wIo.

[57] Thrane G, Sunnerhagen KS, Murphy MA. Upper limb kinematics during the first year after stroke: The stroke arm longitudinal study at the University of Gothenburg (SALGOT). J Neuroeng Rehabil. 2020;17:76. 10.1186/s12984-020-00705-2. 32539738 10.1186/s12984-020-00705-2 PMC7296942

[58] Tomita Y, Rodrigues MRM, Levin MF. Upper limb coordination in individuals with stroke: poorly defined and poorly quantified. Neurorehabil Neural Repair. 2017;31(10–11):885–97. 10.1177/1545968317739998. 29130396 10.1177/1545968317739998

[59] Unger T, De Sousa Ribeiro R, Mokni M, Weikert T, Pohl J, Schwarz A, Held JPO, Sauerzopf L, Kühnis B, Gavagnin E, Luft AR, Gassert R, Lambercy O, Awai Easthope C, Schönhammer JG. Upper limb movement quality measures: Comparing IMUs and optical motion capture in stroke patients performing a drinking task. Front Digit Health. 2024;6:1359776. 10.3389/fdgth.2024.1359776. 38606036 10.3389/fdgth.2024.1359776 PMC11006959

[60] Wang X, Fu Y, Ye B, Babineau J, Ding Y, Mihailidis A. Technology-based compensation assessment and detection of upper extremity activities of stroke survivors: systematic review. J Med Internet Res. 2022;24(6): e34307. 10.2196/34307. 35699982 10.2196/34307 PMC9237771

[61] Wolf SL, Catlin PA, Ellis M, Archer AL, Morgan B, Piacentino A. Assessing wolf motor function test as outcome measure for research in patients after stroke. Stroke. 2001;32:1635–9. 10.1161/01.str.32.7.1635. 11441212 10.1161/01.str.32.7.1635

[62] World Health Organization. International classification of functioning, disability and health: ICF. World Health Organization [Internet]. 2001 [cited 2024 Jun 22]. https://iris.who.int/handle/10665/42407.

