# Supplementary material for: Evaluating inter- and intra-rater reliability in assessing upper limb compensatory movements post-stroke: creating a ground truth through video analysis?
Source: J Neuroeng Rehabil. 2024 Dec 20;21:217. doi: 10.1186/s12984-024-01506-7 (PMC11660698; doi:10.1186/s12984-024-01506-7)
Supplement: Supplementary file 1 — Additional file1 (DOCX 265 KB) [file 12984_2024_1506_MOESM1_ESM.docx]

**Supplemental Material**

**Supplemental Material 1. Labeling System Domains**

The construction of this table is based on items of the Reaching Performance Scale (Levin et al., 2004) and the compensatory movement scoring checklist (Barth et al., 2020).

| **Body Region** | **Movement Direction** | **Description of the characteristics of Movement Compensation** |
| --- | --- | --- |
| ***Trunk*** | Lateral Flexion / Rotation | Excessive trunk side bending on the frontal plane.  Excessive trunk rotation around the longitudinal axis. |
|  | Flexion | Excessive trunk flexion on the sagittal plane. |
| ***Shoulder*** | Flexion | Shoulder flexion occurs with excessive scapular elevation on the sagittal plane.  Lack of shoulder flexion resulting in one or more of the following: shoulder hiking / shoulder abduction / unable to elevate the arm. |
|  | Horizontal adduction / scapula elevation (shoulder elevation) / abduction | Horizontal adduction occurs with excessive scapular protraction or elevation on the frontal plane.  Inadequate abduction and shoulder external rotation when it is required for the task. |
| ***Elbow*** | Extension | Elbow extension is limited or inadequate. |
|  | Flexion | Excessive elbow flexion. |
| ***Forearm*** | Pronation / Supination | Excessive shoulder abduction instead of forearm pronation when pouring, different from unimpaired side Increasing forearm pronation.  Excessive supination in reaching or grasping. |
| ***Wrist*** | Flexion | Excessive wrist flexion during grasp.  Excessive wrist flexion in release of object. |
| ***Fingers*** | Grasp pattern/Hand opening | Use of compensatory grasp pattern.  Inadequate hand opening to grasp the item.  Inadequate hand opening to release the item. |
| ***Global*** | Smoothness | Presence of multiple sub-movements, tremor, dysmetria, jerky movements, segmentation. |
|  | Interjoint Coordination | Temporal cross-correlation of zero-time lag between shoulder flexion and elbow extension. |

Supplemental Material 2, Proposed adjustments to the description of compensatory movement patterns

Based on feedback received on the description of movement compensation characteristics, we propose the adjustments highlighted in red. These modifications aim to enhance the understanding of frequently observed movement patterns in upper extremity motor compensation during a drinking task. The adjusted descriptions are based on the following articles: (Barth et al., 2020; Levin et al., 2004, 2016; Martinez et al., 2020; Thrane et al., 2020; Tomita et al., 2017)

| **Body Region** | **Movement Direction** | **Description of the characteristics of Movement Compensation** |
| --- | --- | --- |
| ***Trunk*** | Lateral Flexion / Rotation | Excessive trunk side bending / rotation around the longitudinal axis. This compensatory movement pattern may occur during the reaching and back transport phases as trunk rotation helps bringing the hand closer to the target on the table. |
|  | Flexion | Excessive trunk flexion on the sagittal plane. This compensatory strategy typically occurs prior to or simultaneously with the initiation of forward hand movement. Trunk flexion may help compensate reduced elbow or shoulder movements. |
| ***Shoulder*** | ~~Flexion*~~ | ~~Shoulder flexion occurs with excessive scapular elevation on the sagittal plane.~~  ~~Lack of shoulder flexion resulting in one or more of the following: shoulder hiking / shoulder abduction / unable to elevate the arm.~~ |
|  | Horizontal adduction / scapula elevation (shoulder elevation) / abduction | Bringing the hand forward during the reaching or back transport phases is initiated with excessive scapula elevation in the frontal plane.  Horizontal adduction ~~occurs~~ with excessive scapular protraction/elevation in the frontal plane: ~~abduction and shoulder external rotation:~~ These compensatory movements ~~when it is required for the task.~~ may occur, while lifting the glass during the forward transport or drinking phases.  Possible reasons for these movement patterns include inadequate shoulder flexion or impairments in elbow movement. |
| ***~~Elbow~~**** | ~~Extension~~ | ~~Elbow extension is inadequate~~ |
|  | ~~Flexion~~ | ~~Excessive elbow flexion~~ |
| ***Forearm*** | Pronation / Supination | ~~Excessive shoulder abduction instead of forearm pronation when pouring, different from unimpaired side Increasing forearm pronation.~~  Excessive supination in reaching or grasping: This compensatory movement pattern may occur during the reaching and transporting phases. To compensate for the loss of finger strength, the forearm is excessively supinated to prevent the glass from falling. |
| ***Wrist*** | Flexion | Excessive wrist flexion during the grasp and release of an object: This compensatory pattern may occur during the reaching and back transport phases, likely to compensate for impaired finger extension.  ~~Excessive wrist flexion in release of object:~~ |
| ***Fingers*** | Grasp pattern/Hand opening | During grasping the glass in the reaching phase, the hand may require multiple adjustments to secure the glass, or the fingers may need to be wind around the glass.  ~~Use of compensatory grasp pattern.~~  ~~Inadequate hand opening to grasp the item~~  ~~Inadequate hand opening to release the item~~ |
| **Global movement characteristics**  *The characteristics listed below are intended for a general analysis of movements across all five phases* | | |
| ***Global*** | Smoothness | Presence of ~~multiple sub-movements,~~ tremor, dysmetria, jerky movements, segmentation.  During the execution of the task, an increased number of movements units may be observed. |
|  | Interjoint Coordination | ~~Temporal cross-correlation of zero-time lag between shoulder flexion and elbow extension.~~  The joints involved in the movement are not synchronized or activated together, resulting in segmented and less fluid movements, causing the hand to deviate from a straight path to reach the object. Primarily, coordination between shoulder and elbow movements is impaired in individuals following a stroke. |

****Shoulder Flexion; Elbow:***  We deleted these items because it pertains to the absence of function rather than the description of a compensatory movement.

**Supplemental Material 3. Statistical details for calculation of ICCs**

To quantify the inter-rater agreement, we used the mean compensation rating of the two ratings from each rater and used the following model (in R notation) in the respective subset:

mean_compensation / 100 ~ (1 | therapist_ID) + (1 | person_ID),

where person_ID is the video number shown to the therapist.

Note, that compensation was divided by 100 to scale the percent values to the interval [0,1] to be aligned with the domain of the beta distribution.

For intra-rater agreement we averaged the ratings of all available raters and used the following model in the respective subset:

mean_from_all_therapists_compensation / 100 ~ (1 | labeling) + (1 | person_ID),

where “labeling” is the first or second instance the same video was shown to the therapist.

To fit the models, we used the R-package brms (Bürkner, 2017) with 10,000 iterations, adapt_delta=0.99, max_treedepth=12, family=zero_inflated_beta() and default priors. Model fit was evaluated with the package performance (Lüdecke et al., 2021) by using posterior

predictive checks.

To quantify ICC consistency (de Vet et al., 2011), we used an adapted version of variance_decomposition() from the package performance:

$$ICC=\frac{{\sigma_{random\_effects}^{2}}}{{\sigma_{random\_effects}^{2}}+{\sigma_{residual}^{2}}}$$

Variances were drawn from the posterior distribution. Credible intervals were defined as 2.5% and 97.5% quantile of the posterior distribution. Values of ICCs or ends of credible intervals outside the interval [0,1] where set to 0 and 1 respectively.

Figures 1-3 show examples of low (Figure 1), medium (Figure 2) and high (Figure 3) disagreement between raters. Disagreement was defined as mean absolute differences of all available ratings averaged over 7 persons (videos shown).

In Figure 1, the combination fingers/grasp/reach was used and had a minimum absolute rating difference of 0 and a maximum absolute rating difference of 7.77.

**
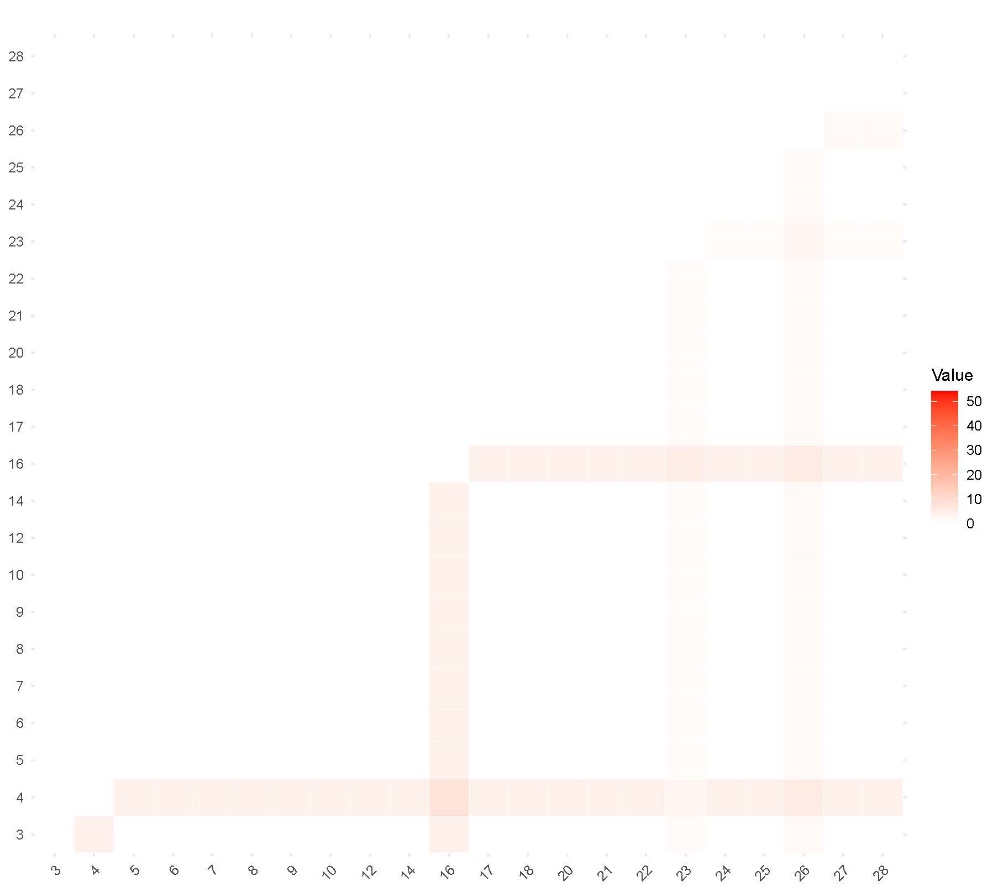
Figure 1.** Low Disagreement.

In Figure 2 the combination shoulder/flexion/return was used and had a minimum absolute rating difference of 0 and a maximum rating difference of 20.43.

**
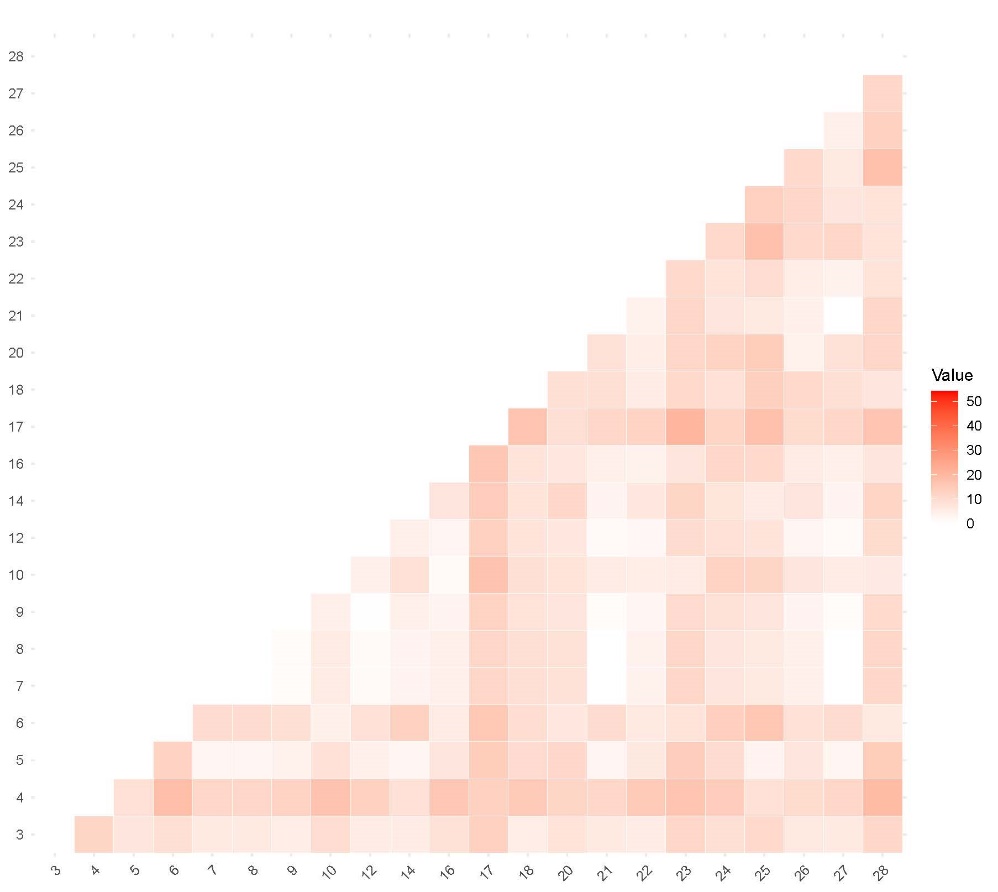
Figure 2.** Medium Disagreement.

In Figure 3, the combination fingers/grasp/reach was used and had a minimum absolute rating difference of 0 and a maximum rating difference of 54.14.

**Figure 3.** High Disagreement.

**
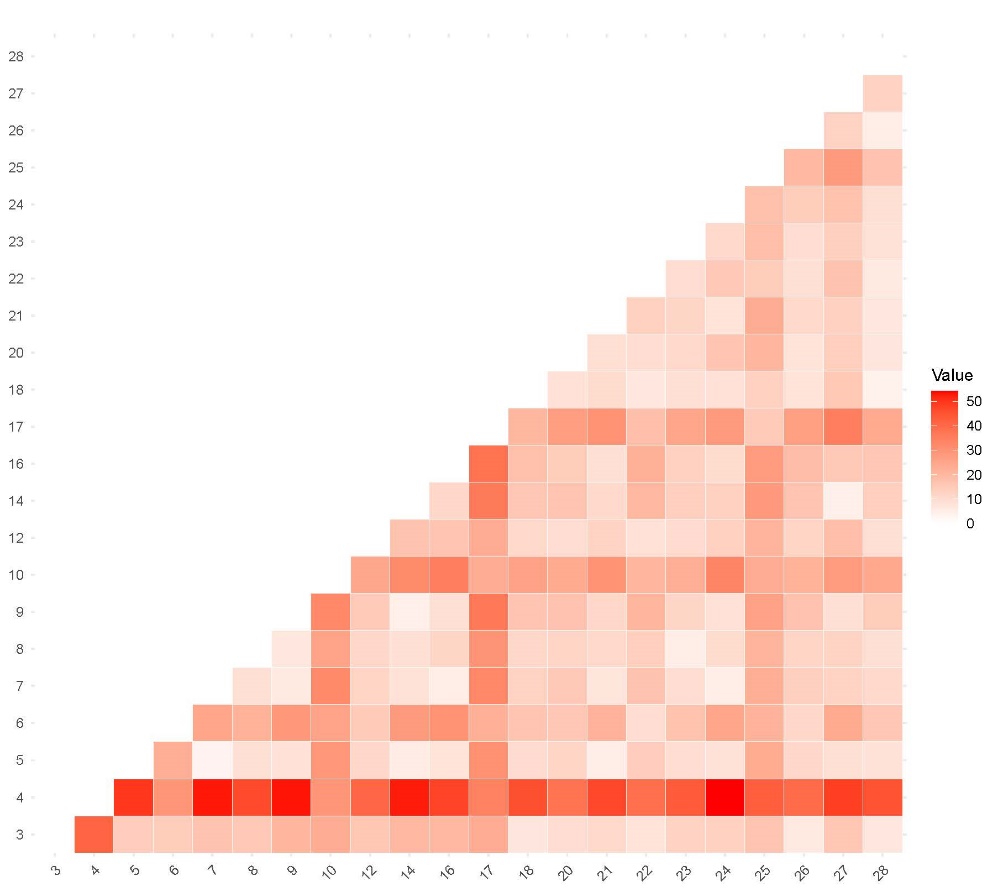
**

The colours were scaled to fit the minimum mean (0) and maximum mean (54.14) over all 55 credible combinations of body region/movement/phases of movement. Missing distance values where imputed using kNN from the package VIM (Kowarik & Templ, 2016).

**Supplemental Material 4. Characteristic Values for deviations from the first to the second labelling round.**

The table presents characteristic values of the deviation in the ratings from the first to the second labelling round.

**Table 2.** Distribution of deviations from the first to the second labelling round

| Movement phases | Min. | 1st quartile | Median | Mean | 3rd quartile | Max. |
| --- | --- | --- | --- | --- | --- | --- |
| Reach | -93 | 0 | 0 | 0.39 | 0 | 79 |
| Transport 1  (Forward transport) | -63 | 0 | 0 | 1.07 | 0 | 100 |
| Drink | -100 | 0 | 0 | 0.11 | 0 | 74 |
| Transport 2  (Back transport) | -100 | 0 | 0 | 0.89 | 0 | 75 |
| Return | -74 | 0 | 0 | 0.45 | 0 | 75 |

Min. = minimum; Max. = Maximum

**References Supplemental Material**

Barth, J., Klaesner, J. W., & Lang, C. E. (2020). Relationships between accelerometry and general compensatory movements of the upper limb after stroke. *Journal of NeuroEngineering and Rehabilitation*, *17*(1), 138. https://doi.org/10.1186/s12984-020-00773-4

Bürkner, P.-C. (2017). brms: An R Package for Bayesian Multilevel Models Using Stanbürkner. *Journal of Statistical Software*, *80*(1). https://doi.org/10.18637/jss.v080.i01

de Vet, H., Terwee, C., Mokking, L., & Knol, D. (2011). *Measurement in medicine: A practical guide*. Cambridge university press.

Kowarik, A., & Templ, M. (2016). Imputation with the R Package VIM. *Journal of Statistical Software*, *74*(7). https://doi.org/10.18637/jss.v074.i07

Levin, M. F., Desrosiers, J., Beauchemin, D., Bergeron, N., & Rochette, A. (2004). Development and Validation of a Scale for Rating Motor Compensations Used for Reaching in Patients With Hemiparesis: The Reaching Performance Scale. *Physical Therapy*, *84*(1), 8–22. https://doi.org/10.1093/ptj/84.1.8

Levin, M. F., Liebermann, D. G., Parmet, Y., & Berman, S. (2016). Compensatory Versus Noncompensatory Shoulder Movements Used for Reaching in Stroke. *Neurorehabilitation and Neural Repair*, *30*(7). https://doi.org/10.1177/1545968315613863

Lüdecke, D., Ben-Shachar, M., Patil, I., Waggoner, P., & Makowski, D. (2021). performance: An R Package for Assessment, Comparison and Testing of Statistical Models. *Journal of Open Source Software*, *6*(60), 3139. https://doi.org/10.21105/joss.03139

Martinez, C., Bacon, H., Rowe, V., Russak, D., Fitzgerald, E., Woodbury, M., Wolf, S. L., & Winstein, C. (2020). A Reaching Performance Scale for 2 Wolf Motor Function Test Items. *Archives of Physical Medicine and Rehabilitation*, *101*(11), 2015–2026. https://doi.org/10.1016/j.apmr.2020.05.003

Thrane, G., Sunnerhagen, K. S., & Murphy, M. A. (2020). Upper limb kinematics during the first year after stroke: The stroke arm longitudinal study at the University of Gothenburg (SALGOT). *Journal of NeuroEngineering and Rehabilitation*, *17*(1), 76. https://doi.org/10.1186/s12984-020-00705-2

Tomita, Y., Rodrigues, M. R. M., & Levin, M. F. (2017). Upper Limb Coordination in Individuals With Stroke: Poorly Defined and Poorly Quantified. *Neurorehabilitation and Neural Repair*, *31*(10–11), 885–897. https://doi.org/10.1177/1545968317739998
